# Immediate action effects motivate actions based on the stimulus–response relationship

**DOI:** 10.1007/s00221-020-05955-z

**Published:** 2020-10-24

**Authors:** Takumi Tanaka, Katsumi Watanabe, Kanji Tanaka

**Affiliations:** 1 Faculty of Arts and Science, Kyushu University, Motooka, Nishi-ku, Fukuoka, Japan; 2 Faculty of Science and Engineering, Waseda University, Shinjuku-ku, Tokyo, Japan; 3 Art and Design, University of New South Wales, Sydney, Australia

**Keywords:** Control feedback, Action effect, Ideomotor theory, Theory of event coding, Sense of agency

## Abstract

The theory of event coding, an influential framework for action planning, suggests that humans first integrate stimulus, response, and action effect into representation (an event file) via their contingencies, and then, the activation of expected action effects drives the associated response. While previous studies have typically examined such functions of action effects after, rather than before or during, the acquirement of the representation, Eitam et al. (Exp Brain Res 229:475–484, 2013a) demonstrated that the presence of immediate feedback to action (i.e., action effects) can instantly elicit faster responses than delayed feedback. However, the underlying mechanism of this faciliatory effect remains unclear. Specifically, while the response–effect relationship has been highlighted, the role of stimuli has not been investigated. To address this issue, the present study conducted four experiments. We first reproduced the faciliatory effects of immediate action effects with between- and within-participants design (Experiments 1 and 2, respectively). Then, we assessed whether immediate action effects facilitate response speed, when stimuli (Experiment 3) and a combination of stimuli and responses (Experiment 4) determined the delay of action effects. The identical response was executed faster when driven by stimuli associated with immediate effects than by those associated with lagged effects. This result indicates that immediate action effects do not reinforce the execution of specific motor actions itself, but facilitate actions depending on the stimulus–response relationship. We discuss the potential mechanism of the facilitation effect.

## Introduction

Our daily activities, such as dialing phone numbers, turning faucets, and switching on lights, are performed to achieve an outcome desired in a certain situation, such as hearing someone’s voice, drinking water, or obtaining clear sight. The theory of event coding, or TEC (Hommel et al. 2001; Hommel 2019), is an influential framework describing the central roles of action effects in action planning. According to the TEC, the representation of actions is composed of the individual features of perceptual effects—such as right/left, fast/slow, and handed/legged—just as that of perceived objects results from a combination of color, shape, location, et cetera. These codes are referenced by both the sensory and motor systems, and are connected via co-activation. For example, if a red traffic light signals a driver to step on the brake to stop a car, the red light, right foot, brake sound, and deceleration codes form a network as one event file (Hommel 2004). Once such an association is established, the activation of the event’s feature codes (e.g., red or slow) spreads to other attributes, eliciting the represented response (e.g., stepping on the brake). This leads us to an interesting observation; subsequent action effects can elicit a causal action process. For example, Hommel (1996) let participants press a key corresponding to a high- or low-pitched tone in the learning phase. Next, in the test phase, these tones were presented simultaneously with visual stimuli, and the participants were asked to respond to the visual stimuli by pressing the keys. Although the tones were task-irrelevant, the participants’ reaction time (RT) was shortest when the presented tone matched the effect of the required response. Such compatibility effects mean that participants acquired the representation of action effects via the learning phase, and thus, its bottom-up activation primed actions in the test phase (Shin et al. 2010). Kiesel and Hoffmann (2004) showed an action can be associated with different effects depending on the preceding stimuli (contexts), and vice versa. This supports the presence of event files based on the stimulus–response–effect (S–R–E) relationship.

The TEC does well in explaining the behavior of action effects after, rather than before or during, acquisition of the representation of action effects (e.g., Elsner and Hommel 2001, 2004; Hommel et al. 2003; Kunde et al. 2004). Elsner and Hommel (2004) revealed that close temporal contiguity between action and effect enhanced the compatibility effect in the test phase, indicating that contiguity may be an important factor in associative development. However, to examine compatibility effects in the test phase, most studies using the two-phase paradigm did not focus on the impacts of action effects on performance in the learning phase. Indeed, although Elsner and Hommel (2004) analyzed the performance during learning and reported the absence of influence by action effects, Karsh et al. (2016) argued that it might result from the insufficient statistical power.

Meanwhile, Eitam et al. (2013a) first demonstrated the instant influence of immediate action effects on actions in the series of actions and effects, referred to as motivation from control, which was later re-named reinforcement from effectiveness by Hemed et al. 2019. In their Experiment 3, participants performed a cued response task that required key presses corresponding to the location of target stimuli, presented at random intervals, as quickly as possible. They pressed the keys faster when the response immediately caused a perceptual change (a white flash during which the targets turned white in color for 100 ms and then disappeared), than when feedback was presented 300 ms or 600 ms after the key was pressed. These effects were observed even when the participants were told that the action effect was irrelevant to the task, as well as when the accuracy of participants' responses was fed back directly, regardless of action effect onsets. The tendency to select responses was also sensitive to control feedback. Participants in Karsh and Eitam’s (2015a) study performed an un-cued response task, with the freedom to choose the key to press, in each trial. They were asked to press each key, at random, and at equal frequency. However, they pressed the keys that caused immediate effects more frequently than the keys with lagged effects, or those without effects. The results provide evidence of the robust influence of action effects, because they were completely independent of the task goal, and actually impeded task performance by biasing participants’ choices. The motivation from control was observed after only a few trials with experience of action effect contingencies (Eitam et al. 2013a; Hemed et al. 2019; Karsh et al. 2016), without the pre-learning.

The motivation from control may reflect the process by which action effects drive the formation of event files (Karsh et al. 2016). To describe the mechanism of motivation from control, Karsh and Eitam (2015b) proposed the control-based response selection (CBRS) framework. According to the CBRS, the presence of action effects can reinforce an action because it is valuable as control feedback evidencing personal control over the environment. Based on the neurocognitive findings that action is represented by the predicted reward value of its effects (e.g., Redgrave et al. 1999; Samejima et al. 2005), Karsh et al. (2016) argued that control values with action effects are also fed back into actions.

**Fig. 1:**
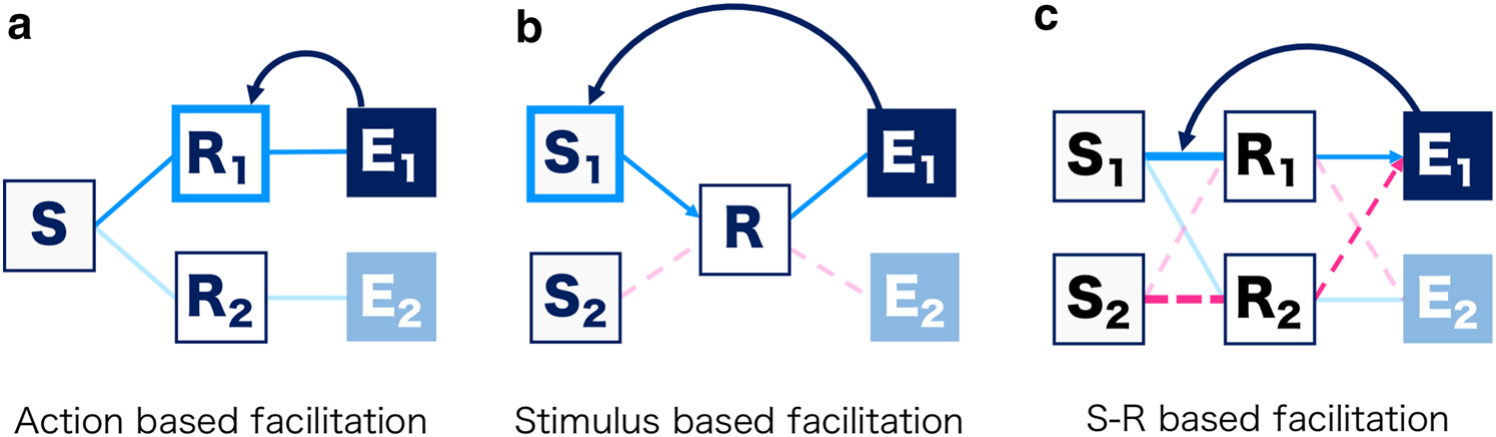
Illustration of the three hypotheses. The capital letter S represents preceding stimuli R represents responding actions, and E represents action effects. Control value from action effects, can facilitate response, by being assigned to **a** actions, **b** stimuli, or **c** the S–R relationships

Although the response–effect (R–E) relationship has been highlighted, the role of stimuli in motivation from control has not been investigated. In the cued response task conducted by Eitam et al. (2013a), the stimuli and responses could not be dissociated because certain actions^1^ were driven by certain types of stimuli (e.g., alignment of a stimulus and response button). On the other hand, in the un-cued response task, the preceding stimuli (e.g., go signal) were always identical and thus completely controlled. Therefore, in the S–R–E relationship, the specific processes reinforced by action effects remain unclear. Considering the possible role of stimulus, we can hypothesize three potential processes underlying motivation from control; action effects can facilitate response, based on (1) actions, (2) stimuli, or (3) a combination of stimulus and response. First, as suggested by CBRS framework, motivation from control may be derived from control-based parameters of each action (Fig. 1a). The second possibility focuses on the role of environmental stimuli (Fig. 1b). The association can be formed between preceding stimuli and consequent rewards, as well as between stimuli and responses (Chen and Kwak 2017). Such positive associations may facilitate human responses to stimuli that predict immediate action effects. This possibility is inconsistent with the finding that action effects mapping induced differences in response speed and frequency, even when all responses were triggered by the same stimuli in un-cued response tasks (Karsh and Eitam 2015a). Nevertheless, it needs to be investigated with the cued task, where participants discriminate the type of preceding stimuli. Note that the first and second possibilities are not theoretically opposed. The control value can be fed back, simultaneously, to both action and stimulus, and either of them may be sufficient to facilitate a response. We treated both possibilities equally in this study. On the other hand, the third possibility integrates the first and second possibilities with S–R relationships. The TEC dictates that the set of preceding stimuli, responses, and effects forms an event file, which then drives response. Given that combinations of stimulus and response differentiate event files and activate specific action effects, action effects may reinforce the response depending on the S–R relationships, and not depending on the independent stimuli or actions (Fig. 1c). Such a case can strengthen the idea that motivation from control compensates for the TEC.

To test the three possibilities, we used cued tasks, which included the factors of actions, stimuli, and S–R relationships, instead of the un-cued tasks that prohibit the manipulation of stimuli. In Experiment 1, we attempted to reproduce the original effect of motivation from control, using a between-participants experiment nearly identical to Eitam et al. (2013a). In Experiments 2, we intended to observe motivation from control in the within-participants cued task, to ascertain that immediate action effects would discriminately fasten specific actions and/or response to specific stimuli. While the within-participants effects have been observed in un-cued task (Karsh and Eitam 2015a), our Experiment 2 was the first investigation in the cued task. Further within-participant experiments were conducted to specify the reinforced process. In Experiments 3, we manipulated the type of stimuli independent of actions. In Experiment 4, we tested whether the facilitation effect would depend on a combination of stimuli and responses. The temporal dynamics in each experiment were also considered to confirm the immediate facilitation of each action effect. As a factor in control feedback, previous studies typically manipulated the contingency (Eitam et al. 2013a; Karsh and Eitam 2015a; Karsh et al. 2016; Penton et al. 2018) or temporal contiguity (Eitam et al. 2013a; Karsh and Eitam 2015a; Karsh et al. 2016) between action and effect. Compared to the contingency, the contiguity appeared less likely to affect explicit knowledge of the degree of control (Karsh et al. 2016). Because this research focuses on the sensorimotor underpinning of motivation from control, only the temporal contiguity was manipulated in the present experiments.

## Experiment 1: Replicating the study of Eitam et al. (2013a)

### Methods

*Participants* Seventy-two adults (34 females; mean age = 20.75 ± 3.16 years) participated in the experiment. As per Eitam et al. (2013a), we calculated a standardized difference (*d*) between the immediate effect, and the longer (600 ms) lagged effect group. An a priori power analysis, using G*Power 3.1 (Faul et al. 2009), revealed that a sample size of 24, for each group, was needed to detect the similar effect size in a two-tailed *t* test^2^ (*d* = 1.08, 95% power, *α* = 0.05).

All participants had normal visual and motor function and were blind to the study objectives. This experiment was approved by the institutional review board of Waseda University (2015-033), and conducted in accordance with the ethical standards of the 1964 Declaration of Helsinki. Written informed consent was obtained from all participants in advance of the research. The statements in this paragraph have been omitted from the methods in the subsequent experiments.

**Fig. 2:**
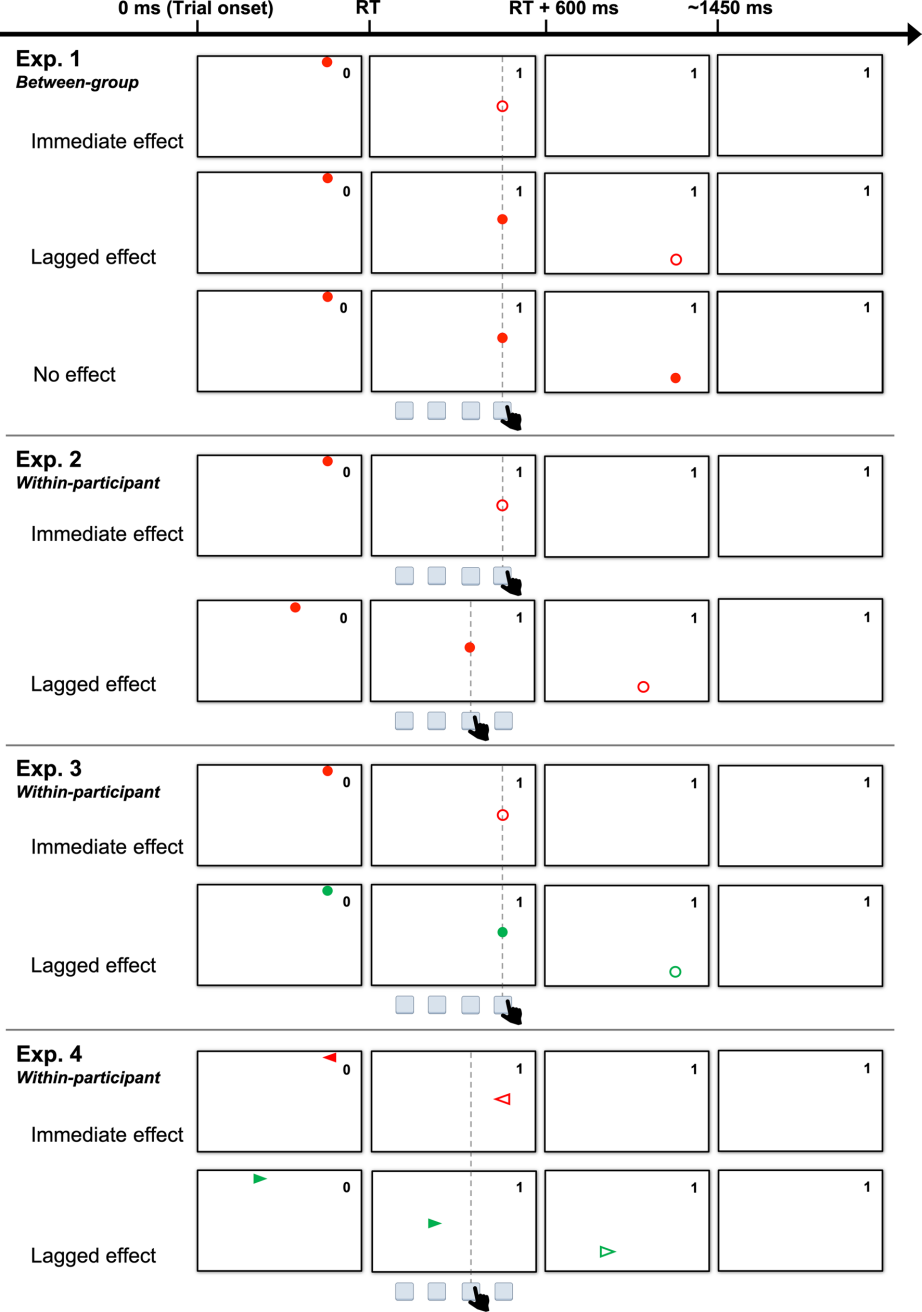
Illustration of trial of each experiment. Participants were asked to press the key corresponding to where the visual target stimulus appeared (Experiments 1, 2, and 3), or the target location and shape combination (Experiment 4), as quickly as possible. Except for the no effect condition in Experiment 1, participant keypresses caused the target stimuli to disappear (represented by translucent stimuli in the figure) immediately (in the immediate condition), or after 600 ms (in the lagged condition). The running score, in the upper right corner of the window, always increased immediately after participants responded correctly. Regardless of RT and response accuracy, each target appeared every 1450 ms

*Materials and procedure* Participants performed a motivation from control task, developed by Eitam et al. (2013a), as shown in Fig. 2. When the task started, a gray game window (16 cm × 27 cm) was displayed in the center of the black screen of a 23-inch LCD monitor (EIZO FORIS FG2421). In each trial, a 2-mm-diameter, red circle (the target stimulus) appeared in one of four horizontal locations, at the top of the window. This target stimulus then descended vertically, and disappeared at the bottom of the window. The speed of the circle was approximately 12.3 cm per second, taking approximately 1300 ms to cross the window. Participants were asked to press one of four keys (d, f, j, k), on a QWERTY keyboard (Apple) as they corresponded to the target location, as quickly as possible. For immediate feedback on the results, which directly indicated task performance, a running score was displayed, in the upper right corner of the game window.^3^ The score increased by one point when the same key was pressed. The time interval for each appearance of the circle was fixed at 1450 ms, irrespective of the participant's response.

For the between-participants manipulation of action effects, participants were randomly assigned to one of three groups: immediate effect, lagged effect, or no effect. The targets disappeared immediately after the participants pressed the key, in the immediate effect group, or after 600 ms in the lagged effect group. For this study, an action effect was defined as the disappearance of a target stimulus, caused by an action. In the group with no effect, the targets did not disappear, and continued descending, despite the participants’ response.

Our motivation was to confirm the robust influence of immediate effects and, therefore, the shorter (300 ms) lagged group, originally included in Eitam et al. (2013a), was omitted. Additionally, two minor changes were made to the original task. First, while the participants' keypresses, in the original task, caused the targets to flash (i.e., the targets turned white for 100 ms after the keypress, and then disappeared), the targets disappeared without changing color in the present task. The change minimized the modulation of attention, due to the saliency of the stimulus, and allowed us to focus on the influence of the effect. Furthermore, action effects occurred, regardless of the accuracy of the keypress, whereas there were no action effects occurred in the error trials of Eitam et al. (2013a). For example, after the target stimulus was displayed in the far left, corresponding to the “d” button, the action effects still occurred if a participant pressed the “f” button. This allowed us to separate control feedback, from the task-related outcome feedback.

The participants engaged in 12 experimental blocks consisting of 40 trials, for a total of 480 trials. All the experiments were controlled using MATLAB (The Math Works, Natick, MA) with Psychotoolbox (Brainard 1997; Kleiner et al. 2007; Pelli 1997) using a MacMini (Apple).

## Results and discussion

The main dependent variables were the median RT and correct response rate for each block. The median is robust to outliner data (Whelan 2008), and therefore, a previous study analyzed medians instead of means of RTs (Penton et al. 2018).

Data of median RT and correct response rate were excluded from analyses if they were outside of two standard deviations from the group mean. We removed one participant from the immediate effect group, two from the lagged effect group, and one from the no effect group (4/72 =  ~ 6% participants in total). Only RTs from successful trials were analyzed, where participants pressed the correct key. The open-source statistical software, *jamovi* (The jamovi project 2019), was used for the analyses of variance (ANOVA), calculation of effect size, with partial *η*^2^, and visualization. To evaluate the degree that our data support the null hypothesis (H_0_) or alternative hypothesis (H_1_), we also ran Bayesian ANOVAs using the default Cauchy prior (*r* scale 0.5 for fixed effects, 1 for random effects) in jamovi, and reported Bayes factor based on Bayesian model averaging (BF_Inclusion_) that represents changes of odds by including a certain effect into the model (JASP Team 2018; Morey and Rouder 2018; Rouder et al. 2012). We interpreted BF_Inclusion_ based on traditional conventions (Jarosz and Wiley 2014; Jeffreys 1961); 3–10 (0.33–0.10) represents substantial, 10–30 (0.10–0.05) represents strong, 30–100 (0.05–0.01) represents very strong, and > 100 (< 0.01) represents decisive evidence for H_1_ (H_0_).

**Fig. 3:**
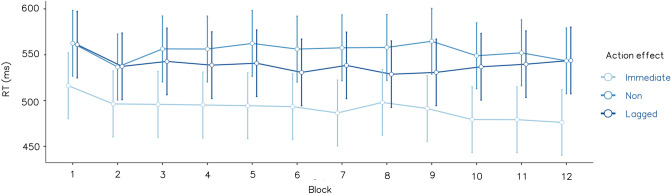
Reaction time averaged by action effect groups and blocks, from Experiment 1. Error bars indicate standard error. The response was significantly faster when action effects immediately followed responses, than when lagged or no effects were displayed, throughout the experiments (*p* < 0.05)

A two-way mixed ANOVA was run on median RTs, with a between-participants factor for action effects (i.e., immediate, lagged, and no effect) and a within-participant factor for the block (block 1 to block 12). Only the main impact of the action effect was significant (*F*(2, 65) = 4.514, *p* = 0.015, partial *η*^2^ = 0.122, BF_Inclusion_ = 3.868). The participants in the immediate effect group reacted faster than those in the lagged (*t*(65) = 2.151, adjusted *p* = 0.035), and no effect (*t*(65) = 2.887, adjusted *p* = 0.016) groups. There was no significant difference between the lagged and no effect groups (*t*(65) = 0.703, *p* = 0.484). The main impact of block (*F*(11, 715) = 1.383, *p* = 0.176, partial *η*^2^ = 0.021, BF_Inclusion_ = 0.012) and the interaction (*F*(22, 715) = 0.570, *p* = 0.943, partial *η*^2^ = 0.017, BF_Inclusion_ < 0.01) were not supported. Figure 3 shows the mean of median RTs in Experiment 1.

The same type of two-way mixed ANOVA was run on the correct rate (see Table 1). Although the results showed a significant main effect of block (*F*(11, 715) = 3.000, *p* < 0.001, partial *η*^2^ = 0.044, BF_Inclusion_ = 5.902),^4^ there was no significant main effect of action effect (*F*(2, 65) = 2.489, *p* = 0.091, partial *η*^2^ = 0.071, BF_Inclusion_ = 0.540) nor the significant interaction (*F*(22, 715) = 1.135, *p* = 0.303, partial *η*^2^ = 0.034, BF_Inclusion_ = 0.015). The absence of the influence of action effects prevented an explanation of the speed–accuracy trade-off for the faster responses in the immediate effect group.

**Table 1:** Correct rates (%) by action effect conditions and blocks from Experiment 1

Condition	Block												
	1	2	3	4	5	6	7	8	9	10	11	12	Overall
Immediate effect	91.7	94.2	94.0	92.8	93.8	94.2	94.7	94.8	93.7	93.7	93.4	93.7	93.7
Lagged effect	86.9	92.6	95.9	96.3	93.3	92.8	91.8	91.8	94.7	90.7	92.4	93.0	92.7
No effect	93.6	95.7	97.0	96.8	97.4	96.1	96.2	95.8	96.8	95.1	94.5	91.7	95.6

In summary, the benefit in performance from an immediate effect was successfully reproduced in Experiment 1. The effect appeared to be robust and independent of the outcome feedback. Because the facilitation was observed even when all the alternative actions caused the same immediate, or lagged, action effect, the explanation that salient action effects may provide cues to identify and select associated actions does not hold. Contrarily, the results supported the idea that control feedback has an absolute motivational value and improves task performance. Based on the results of Experiment 1, we conducted the following three within-participants design experiments to explore what specific processes would be facilitated by immediate action effects.

## Experiment 2: Impacts of action effects based on action

### Methods

*Participants* Twenty-four adults (10 females; mean age = 20.75 ± 1.66 years) were newly enrolled for Experiment 2. Based on *d,* between the immediate and lagged effects in Experiment 1, an a priori power analysis using G*Power 3.1 (Faul et al. 2009) revealed that a sample size of 22 was needed, to detect the similar effect size in a one-tailed paired *t* test (*d* = 0.56, 80% power, *α* = 0.05). A couple of participants, per group (*n* = 24), were removed from the analyses in Experiment 1. Therefore, we eventually recruited a total of 24 participants.

*Materials and procedure* The experimental task was basically the same as Experiment 1, except for the following action effect manipulation. Instead of the between-participants manipulation used in Experiment 1, the action effect factor was manipulated in a within-participant manner in Experiment 2. Two out of four response keys were assigned to the immediate effect condition, and the remaining keys were assigned to the lagged effect condition. The target disappeared immediately after pressing the former, and 600 ms after pressing the latter. Our focus was on the effects of temporal contiguity; therefore, the no effect condition was neither implemented in Experiment 2, nor in the subsequent experiments.

Participants performed six experimental blocks with a total of 80 trials, resulting in 480 trials. The combinations of keypresses and action effects (e.g., d or f key triggered the immediate condition, and j or k key triggered the lagged condition) were counterbalanced between participants; one of the six (= 4(4–1)/2) possible combinations was assigned to each participant.

## Results and discussion

Based on the same criteria as Experiment 1, the data from four participants were excluded from further analysis (4/24 =  ~ 17%). The RTs of incorrect responses were also removed.

**Fig. 4:**
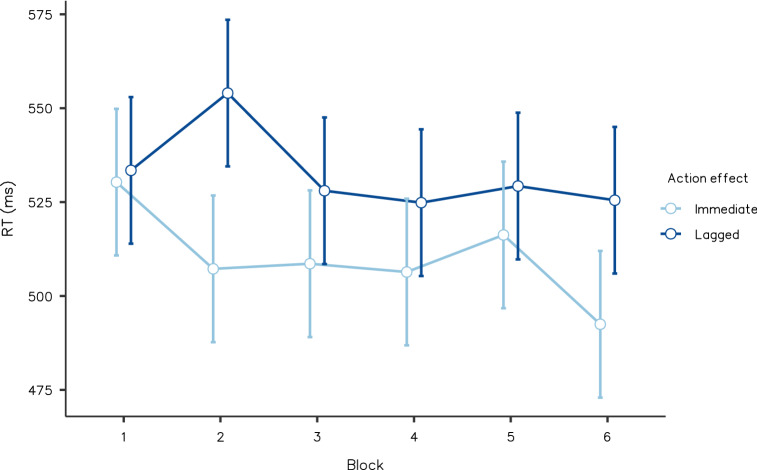
Reaction time averaged by action effect conditions and blocks in Experiment 2. Error bars indicate standard error. Responses with keypresses that caused immediate effects were significantly faster than responses with keypresses that were followed by lagged effects (*p* < 0.05)

For median RTs, we conducted a 2 (immediate and lagged action effects) × 6 (blocks one to six) within-participants ANOVA. We found that action-based action effects had a strong main impact (*F*(1, 19) = 7.049, *p* = 0.016, partial *η*^2^ = 0.271, BF_Inclusion_ = 16.126), where the participants pressed the keys that caused immediate effects, more rapidly than the keys that caused lagged effects. Neither the main impact of the block (*F*(5, 95) = 1.126, *p* = 0.352, partial *η*^2^ = 0.056, BF_Inclusion_ = 0.057) nor the interaction (*F*(5, 95) = 0.860, *p* = 0.511, partial *η*^2^ = 0.043, BF_Inclusion_ = 0.081) were significant. Figure 4 shows the mean RTs in Experiment 2.

The same two-way ANOVA with correct rates also showed a significant main impact of action effect (*F*(1, 19) = 5.343, *p* = 0.032, partial *η*^2^ = 0.220, BF_Inclusion_ > 100). Note that the action effect factor here corresponded to the actual actions performed, and not the target locations (to-be-executed actions). Therefore, the results were interpreted differently from the other experiments. The actions associated with immediate effects had more room for human error, even when the targets required actions with lagged effects (see Table 2). The main impact of block (*F*(5, 95) = 1.095, *p* = 0.368, partial *η*^2^ = 0.055, BF_Inclusion_ = 0.062) and the interaction (*F*(5, 95) = 1.015, *p* = 0.413, partial *η*^2^ = 0.051, BF_Inclusion_ = 0.060) were not significant.

**Table 2:** Correct rates (%) by action effect conditions and blocks from Experiments 2, 3, and 4

Condition	Block						
	1	2	3	4	5	6	Overall
Experiment 2*							
Immediate effect	92.97	93.73	95.30	94.54	92.83	92.77	93.69
Lagged effect	94.97	95.90	96.01	96.83	95.05	97.01	95.96
*Experiment 3*							
Immediate effect	94.51	95.80	95.62	95.85	94.81	95.15	95.29
Lagged effect	95.82	95.87	95.36	94.73	92.91	95.62	95.05
*Experiment 4*							
Immediate effect	90.44	93.92	91.08	93.49	94.03	93.46	92.74
Lagged effect	89.99	92.23	93.69	92.40	92.65	94.66	92.60

*A significant difference at the .05 probability level, between action effect conditions

The results of Experiment 2 indicated that immediate action effects could selectively reinforce a specific stimulus-driven response in the cued task. The participants executed actions with immediate effects more quickly, and even impulsively. Such tendencies appeared to be consistent with the prediction from the CBRS framework; reinforcement from immediate action effects may be assigned to the actions that caused them.

However, in Experiment 2, we could not specify what processes were reinforced by immediate effects. Because the actions performed by participants corresponded to the stimulus features (i.e., locations), we could not differentiate between the facilitation of particular actions (movements) and that of response to particular stimuli. There remained another possibility that action effects could modulate the processing of specific stimuli or their features. Therefore, we manipulated the type of stimuli independent of actions in the next experiment.

## Experiment 3: impacts of action effects based on stimulus

### Methods

*Participants* Twenty-four adults (14 females; mean age = 20.79 ± 1.89 years) were newly enrolled. The sample size was decided in the same way as Experiment 2.

*Materials and procedure* The experimental task was basically identical to Experiment 1, except for the manipulation of action effects. In Experiment 3, the target stimuli (i.e., descending circles) were red or green. The two types of targets were randomly displayed in each location, with equal frequency. Instead of between-participants manipulation, the action effect condition depended on the color of targets within-participants. Keypresses for the targets with one color caused immediate effects, and those for the other color caused lagged effects.

Participants performed six experimental blocks of 80 trials, resulting in 480 trials. The combinations of colors and action effects (e.g., red for immediate effects and green for lagged effects) or the reverse were balanced between participants.

## Results and discussion

**Fig. 5:**
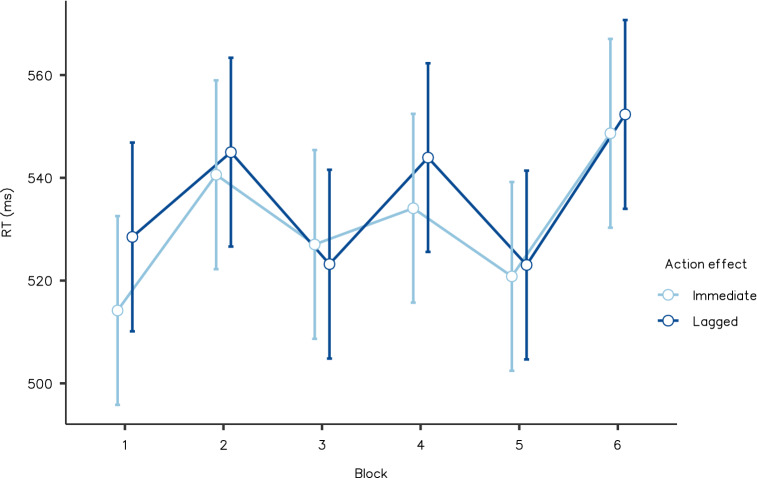
Reaction time averaged by action effect conditions and blocks, from Experiment 3. Error bars indicate standard error. There was no significant difference in response rates between response to stimuli contingent with immediate effects, and response to stimuli contingent with lagged effects

Based on the same criteria as the preceding experiments, the data from four participants were excluded from further analyses (4/24 =  ~ 17%). We conducted a 2 (immediate and lagged action effects) × 6 (blocks one to six) within-participants ANOVA for median RTs. Our data substantially supported the absence of main impact of action effect (*F*(1, 19) = 1.040, *p* = 0.321, partial *η*^2^ = 0.052, BF_Inclusion_ = 0.187). The main impact of block (*F*(5, 95) = 1.317, *p* = 0.264, partial *η*^2^ = 0.065, BF_Inclusion_ = 0.840) and the interaction (*F*(5, 95) = 0.943, *p* = 0.457, partial *η*^2^ = 0.047, BF_Inclusion_ = 0.030) were not significant. The averaged median RTs in Experiment 3 are shown in Fig. 5. The two-way ANOVA for the correct rate showed no significance (action effect: *F*(1, 19) = 0.233, *p* = 0.635, partial *η*^2^ = 0.012, BF_Inclusion_ = 0.155; block: *F*(5, 95) = 0.936, *p* = 0.461, partial *η*^2^ = 0.047, BF_Inclusion_ = 0.078; interaction: *F*(5, 95) = 1.367, *p* = 0.244, partial *η*^2^ = 0.067, BF_Inclusion_ = 0.093; See Table 2 for details).

The stimulus features associated with immediate effects (color) did not influence performance, rejecting our second hypothesis that motivation from control is the selective facilitation of response to specific stimuli contingent with control feedback (Fig. 1b). There remained the third possibility that motivation from control occurs based on the S–R relationship (Fig. 1c), as well as the possibility of action-based facilitation (Fig. 1a). It should be noted that the cue stimuli included two independent features—location and color. The target feature associated with action effects (color) was task-irrelevant; Participants needed to distinguish the cue locations, but not colors, to select a response. Thus, while the association might be formed between target color and action effect, it was separated from the S–R relationship between target location and response. Action effects may reinforce responses by being mapped into the S–R relationship. Experiment 4 tested this possibility.

## Experiment 4: impacts of action effects based on stimulus–response

### Methods

*Participants* Twenty-four adults (19 females; mean age = 21.08 ± 4.59 years) were newly enrolled.

*Materials and procedure* The task in Experiment 4 was also based on the motivation from control task. In each trial, a red triangle pointing to the right (i.e., a right arrow) or a green triangle pointing to the left (i.e., a left arrow) appeared and descended as targets. Participants were asked to press the key that corresponded to the next location, in the direction from which the target appeared. For example, if the right arrow was displayed in the far left, participants had to press the second key from the left. Importantly, the action effects were direction (and color) dependent.^5^ Responses to targets in one direction caused an immediate effect, whereas responses to targets in the other direction caused lagged effects. Thus, each participant experienced either immediate effect or lagged effect, after pressing one of the middle two keys. This enabled the assessment of the impacts of immediate effects, depending on the combination of stimulus and response. If stimuli associated with immediate effects elicited shorter RTs than those with lagged effects, even when the same key was pressed, the effects cannot be explained by action-based facilitation alone, but should be considered the S–R relationship.

The right arrow was never displayed on the right-most side, and the left-most arrow was never displayed on the left side. Therefore, there were six types of targets, with specific direction and location: a right arrow in three locations on the left, and a left arrow in three locations on the right. They were randomly displayed 15 times per block. Participants completed six blocks, resulting in a total of 540 trials. The combinations of target type and action effect (e.g., right/red for immediate effects, and left/green for lagged effects) were counterbalanced among the participants.

## Results and discussion

The data from three participants were excluded from further analyses based on the same criteria (3/24 =  ~ 13%). The RTs of incorrect responses were removed. The RTs of the far right and far left keypresses were not analyzed, because they were made only when left or right arrows were displayed, respectively, and did not fit with the motivation for the experiment.

**Fig. 6:**
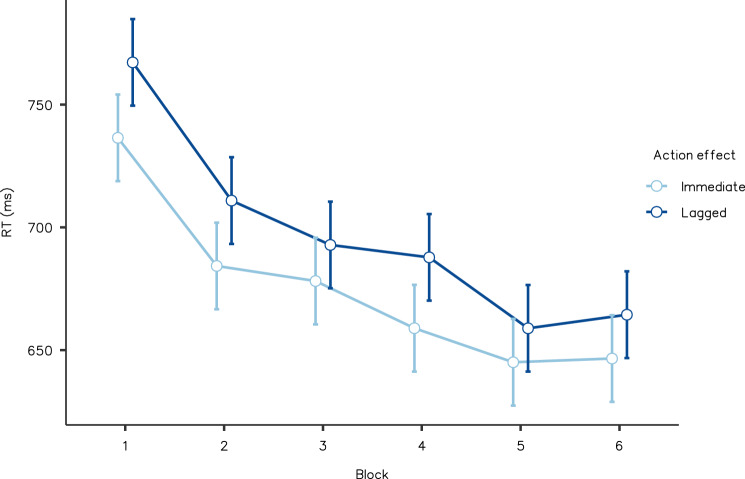
Reaction time averaged by action effect conditions and blocks, from Experiment 4. Error bars indicate standard error. Responses when actions to certain stimuli predicted immediate effects were faster than when they predicted delayed effects (*p* < 0.05)

We conducted a 2 (immediate and lagged action effects) × 6 (blocks one to six) within-participants ANOVA, for median RTs. There was strong evidence for the main impact of action effect (*F*(1, 21) = 4.718, *p* = 0.042, partial *η*^2^ = 0.191, BF_Inclusion_ = 58.940); participants in the immediate effect condition reacted faster than those in the lagged condition. The main impact of block (*F*(5, 100) = 17.537, *p* < 0.001, partial *η*^2^ = 0.467, BF_Inclusion_ > 100) was also supported. There was no significant interaction (*F*(5, 100) = 0.845, *p* = 0.521, partial *η*^2^ = 0.041, BF_Inclusion_ = 0.031). The mean RTs in Experiment 4 are shown in Fig. 6. The two-way ANOVA for correct rate showed no significance (action effect: *F*(1, 20) = 0.014, *p* = 0.908, partial *η*^2^ < 0.001, BF_Inclusion_ = 0.142; block: *F*(5, 100) = 1.637, *p* = 0.157, partial *η*^2^ = 0.076, BF_Inclusion_ = 0.290; interaction: *F*(5, 100) = 1.516, *p* = 0.192, partial *η*^2^ = 0.071, BF_Inclusion_ = 0.076; See Table 2 for details).

Despite the absence of effects in Experiment 3, the stimulus feature associated with control feedback (i.e., color/shape) played a critical role in forming motivation from control in Experiment 4. The identical actions were performed reliably faster when they were driven by stimuli associated with immediate effects, than when driven by stimuli associated with lagged effects. The actions caused immediate and lagged action effects, with exactly equal frequency, throughout the experiment. Therefore, the superior processing of action with high control value (Fig. 1a) cannot explain such contextual facilitation. The results supported the possibility that the features mapped to the S–R relationship can activate the associated control feedback and facilitate action.

Another possibility is that the difference in task difficulty could have caused the differential results of Experiments 3 and 4. In fact, the average RT of median in Experiment 3 was approximately 150 ms shorter than in Experiment 4. However, although the RT decreased in accordance with the blocks, indicating that participants became gradually accustomed to the task, the impact of action effects was not modulated by the blocks. To examine the relationship between the influence of temporal contiguity and difficulty, a correlation was calculated in Experiment 4 between the average RT of each participant and the differences between conditions (RT in the lagged condition—RT in the immediate condition). There was no significant correlation (*t*(18) = 0.122, *r* = 0.028, *p* = 0.904), indicating the benefit from immediate effects did not depend on the task difficulty. This was the same in the other experiments (*t*(18) = 0.004, *r* < 0.001, *p* = 0.997 in Experiment 2; *t*(18) = 0.915, *r* = 0.211, *p* = 0.372 in Experiment 3). Therefore, task difficulty was not a critical modulator of response facilitation with immediate effects.

## General discussion

This study investigated how immediate action effects facilitate responses, via the experience of simple action effect contingencies. Specifically, we assessed three possible processes in which action effects facilitate response, based on processing of actions, stimuli, or the S–R relationship.

Our results revealed that the response facilitation could not be explained by independent processing of the actions or stimuli. In Experiment 1, the robust facilitating effect was reproduced. The participants who received immediate feedback, reacted faster than the participants who received lagged feedback, or no feedback. Experiment 2 with the within-participants design ensured that immediate effects can selectively fasten processing for particular actions and/or stimuli. The RT for actions causing immediate effects was shorter than those causing lagged effects. The stimulus-based process was rejected in Experiment 3. The target features, associated with the control feedback, did not elicit response facilitation. However, Experiment 4 provided evidence that stimuli do play a role in motivation from control. The identical actions were performed faster when driven by stimuli associated with immediate effects, than when driven by stimuli associated with lagged effects.

These results implicate that motivation from control may result from more complex processes than previously thought. The CBRS framework assumes that the control values are assigned to the action that caused the effect, resulting in response facilitation (Karsh and Eitam 2015b). This predicts the motor parameters of certain response are basically fixed by its own values. The fact that response speed for identical actions either accelerated or decelerated in response to the trigger stimulus is contrary to such a prediction. A potential interpretation is that the rewarding value of immediate action effects can be assigned to, and reinforce, S–R associations. Based on TEC, the action effect should be represented with the stimulus and response in an event file as the associations of individual features. The control value may promote development or activation of the event file, thereby resulting in faster responses. Given the absence of modulation by time series, motivation from control may be able to contribute to the early stage of event file formation. On the other hand, when a stimulus feature associated with control feedback (color) was separated from the responses, and thus, from the event files (Experiment 3), the facilitation did not occur. The S–R dependence of motivation from control provides new evidence for the role of the rewarding value of action effects in developing event files.

An explanation based on higher cognitive processes should be considered as well. Substantial studies have claimed that an event is perceived as one’s own action effect by predicting the action in advance (comparator model; e.g., Blakemore et al. 1999; Wolpert et al. 1998). Therefore, predictability has been considered a crucial factor of motivation from control (Karsh et al. 2016). Nevertheless, our Experiment 3 showed that the immediate effects do not always motivate responses when the action effects are predictable. This may be because the predictive cue of action effects (color) was task-irrelevant, and therefore filtered by selective attention (Eitam et al. 2013b; Wyble et al. 2019). In this context, our results add evidence that action effects must be not only predictable but also actually predicted in order to facilitate responses. While a similar prediction (pre-activation) function is also implemented in TEC (Hommel 2019), it is unclear whether the prediction underlying motivation from control is independent from the event file. To investigate this, one may be able to use a dual-task paradigm in which participants must attend to cues that predict action effect, but are irrelevant to the current response.

At this point, we cannot fully specify the regulatory mechanism of motivation from control depending on the S–R relationship. Focusing on the sensorimotor processes, we did not measure cognitive processes such as prediction and attention. Moreover, our manipulation of contiguity might also influence explicit knowledge of the degree of control. Although Karsh et al. (2016) manipulated the contiguity in four step (0 ms, 150 ms, 300 ms, and 450 ms) and showed no impact on explicit judgment of agency, the difference between 0 and 600 ms in this study may have been more salient for participants. CBRS suggested that explicit knowledge biases the action selection (i.e., conducting actions that one believes is more likely to cause effects) independently of sensorimotor modulation of action execution (Karsh and Eitam 2015b). As a tangible reward (money) did not influence the speed of responses in the cued task, the motor level facilitation might result through a process different from the inverse model, in which an action was selected by their predicted value (Karsh et al. 2020). In our Experiment 2, participants were more likely to execute incorrect responses causing immediate effects. Although this supported the rewarding function of action effects, it deviated from the facilitation based on the S–R relationship. The increase of error responses implicates the possibility of top-down modulation in our experiments, as well as that of the speed–accuracy trade-off. Future research should investigate how the S–R–E relationships contribute to speed and selection of actions in un-cued tasks, in which a single stimulus is associated with different actions.

In conclusion, the present study demonstrated that immediate action effects as control feedback facilitate a response depending on the S–R relationship. Humans can automatically improve their responsiveness to certain stimuli, via the experience that their actions affect the environment. Such S–R-based facilitation may contribute to the acquisition of flexible and complex behavioral patterns. Based on the TEC, action effects have the potential to be useful in helping to form associations between stimuli, responses, and action effects, and to integrate into the representation of the event files that govern our perceptions and actions.

## Data Availability

The datasets analyzed in this study are available in the Open Science Framework repository, https://osf.io/va5uw/?view_only=6942fe5f84444f328d8d73695e72228f.

## Footnotes

1 In this paper, we used the term “action” as a specific movement regardless of what drives it, and used the term “response” as a specific action that is executed in response to a specific stimulus.

2 Only a two-way mixed ANOVA was conducted in this experiment, and because the comparison requires critical evaluation, the power was calculated by assuming a *t* test between the immediate and lagged conditions.

3 A reviewer pointed out that the running score should have been placed in the middle of the screen. The assignments of action effects to keys (cues) were counterbalanced, and thus the position of the score did not have systemic impacts on the results; yet, we think the advice will improve our future research.

4 The theoretical interest of this study, for the sake of conciseness, is the effect of motivation from control, and this article focuses on describing the main impacts of action effects, and the influence of interaction between action effects and the blocks, not the detail of the main impacts of the blocks, i.e., only the main effects.

5 Although the color and the direction were both sufficient for response selection, and action effect manipulation, both features were changed to allow participants to easily distinguish the target types, and to minimize the difference in task difficulty, among the experiments.

## Code availability

The jamovi sources for analyses during the current study are available in the Open Science Framework repository, https://osf.io/va5uw/?view_only=6942fe5f84444f328d8d73695e72228f.
